# Medicaid professional fees for treatment of opioid use disorder varied widely across states and were substantially below fees paid by medicare in 2021

**DOI:** 10.1186/s13011-022-00478-y

**Published:** 2022-07-06

**Authors:** Lisa Clemans-Cope, Victoria Lynch, Maya Payton, Joshua Aarons

**Affiliations:** 1 Health Policy Center at Urban Institute, 500 L’Enfant Plaza SW, Washington, DC 20024 USA; 2 Economics at UC San Diego, 9500 Gilman Dr. #0508, Mail Code: 0508, La Jolla, California, 92093 USA

**Keywords:** Opioid use disorder, Medicaid, Medicare reimbursement, State policy, Federal policy, Methadone treatment

## Abstract

**Background:**

As Medicaid is the largest payer for opioid use disorder (OUD) treatment services in the United States, information about Medicaid provider reimbursement is critical, and Medicaid payment policies influence the structure of OUD treatment services for everyone with OUD treatment needs.

**Methods:**

We collected Medicaid professional fees for OUD treatment and related services for the District of Columbia and fifty state Medicaid programs and the Medicare program in 2021. We create three fee indexes related to OUD treatment, with an emphasis on services related to first-line medication treatments in outpatient settings. We then create Medicaid fee indexes and Medicaid-to-Medicare fee indexes.

**Results:**

Weekly Medicaid fee bundles for methadone treatment at OTPs in 2021 varied widely, more than 4-fold across states. The Medicaid-to-Medicare fee index shows that the national average Medicaid fee bundle was 56 percent of Medicare fees for regular methadone treatment at OTPs in 2021. For services related to OUD treatment, Medicaid fees varied up to 5-fold and larger across the components of each of the four services, and Medicaid fees were low relative to Medicare for almost all state services examined. The Medicaid-to-Medicare fee index was 64 percent of Medicare fees in 2021, ranging from 52 percent for evaluation & management to 76 percent for toxicology testing.

**Conclusions:**

There appears to be little justification for such large variation in Medicaid fees across states. In addition, the generally low fees in Medicaid persist despite recent efforts to increase access to opioid use disorder treatment for Medicaid enrollees, and have important implications for access to life-saving treatment during the current opioid overdose crisis.

**Supplementary Information:**

The online version contains supplementary material available at 10.1186/s13011-022-00478-y.

## Background

As COVID-19 infections and mortality raged across the globe in 2020, the epidemic of opioid overdose deaths tragically accelerated in the United States [1, 2]. First-line treatments for opioid use disorder (OUD), methadone and buprenorphine, significantly decrease opioid overdose mortality and opioid-related morbidity, but are dramatically underutilized [3]. However, the drivers of underutilization of effective treatments for OUD are not well understood, including whether provider payments are adequate to ensure access to OUD treatment and how variation in provider payments may relate to disparities in SUD treatment. As Medicaid is the largest payer for OUD treatment services in the United States, [4] information about Medicaid provider reimbursement is critical, and Medicaid payment policies influence the structure of OUD treatment services for everyone with OUD treatment needs. Thus, examining Medicaid reimbursement for OUD treatment can inform policy conversation about the extent to which reimbursement is a lever to expand access and to shape access through alternative payment systems and payment model reform. To our knowledge, state Medicaid reimbursement rates for OUD treatment services have not been previously collected across state programs and no systematic information is available on how they compare to reimbursement rates set by other payers such as Medicare. This study aims to fill this knowledge gap.

Previous research examining Medicaid physician fees across a variety of types of care found that in 2019, as in prior years, Medicaid reimbursement was below Medicare and private insurance fees [5]. While no study has examined Medicaid fees related to OUD treatment, several recent changes have affected the use of these services in Medicaid. First, Medicaid coverage of substance use benefits and Medicaid’s enrollment of populations with disproportionate burden of OUD grew substantially under the Affordable Care Act (ACA) [6, 7]. Secondly, state and federal policy makers have implemented policies expanding coverage for and access to Medicaid services for OUD treatment through: increasing OUD treatment benefits beyond those required by the ACA, [8] reforming delivery systems including Opioid Health Homes [9] and increasing the number of providers trained to treat OUD, [10] and, in some states, increasing provider reimbursement in Medicaid for OUD treatment services [11]. Low reimbursement rates in state Medicaid programs have been found to correlate with lower rates of providers accepting new Medicaid patients, which may reduce access to care for enrollees [12].

This article presents the first examination of Medicaid professional fees related to the treatment of OUD to assess how Medicaid fees compared with Medicare fees in 2021 across states and services. This study is limited to fee-for-service fees, which is not ideal given Medicaid’s reliance on managed care, however, Medicaid managed care organizations’ fees are largely unavailable for research purposes. We find that Medicaid professional fees for treatment of OUD varied widely across states and were substantially below fees paid By Medicare. In various initiatives across states in the past years, several states increased Medicaid fees for services related to the treatment of OUD [11]. These fee increases were, in part, attempts to address long-standing concerns that low Medicaid physician fees for effective or recommended services may impede access to care for enrollees with OUD. Our analysis aims to provide a baseline for evaluating the effects of variation across Medicaid in these fees.

## Methods

We collected Medicaid professional fees for OUD treatment and related services by building on a survey of fee-for-service physician fees for common procedures in the District of Columbia and fifty state Medicaid programs which has been conducted since 1993 [5]. In this study, we collected new data to create three indexes related to OUD treatment in state Medicaid programs in 2021, with an emphasis on services related to first-line medication treatments in outpatient settings. The three indexes are: (1) a methadone bundle of services standardized to the components of Medicare’s Opioid Treatment Program (OTP) weekly service bundle [13], (2) a methadone bundle plus additional care coordination, case management, Opioid Health Home or Center Of Excellence program services, and (3) four other services related to OUD treatment (evaluation and management, psychotherapy, toxicology testing, and substance use screening and psychiatric diagnostic evaluation). We create a Medicaid fee index for each of these three service indexes, and a Medicaid-to-Medicare fee index for the regular methadone bundle and the other services related to OUD treatment. This data has been made publicly available [14].

Medicare payments for OUD treatment and related services are a relevant comparison for Medicaid for several reasons. Medicare enrollees have substantial rates of OUD and OUD treatment [15, 16]. In addition, the Medicare rate for the regular methadone bundle was recently set based on a review of services and reimbursements by other payers such as TRICARE and some state Medicaid programs [17], facilitating the construction of the standardized bundle in this study. Lastly, Medicare’s geographic adjustments, which adjust provider payments to reflect the local costs of providing care, facilitate a better comparison of payments across states.

To select a set of procedure codes for tracking Medicaid reimbursement for OUD treatment across state Medicaid programs, we started with a broad list of approximately 50 codes related to OUD treatment. This initial code list were Healthcare Common Procedure Coding System (HCPCS) and Current Procedural Terminology (CPT) codes that we identified as capturing the continuum of care of effective OUD treatment as defined by the American Society of Addiction Medicine (ASAM) [18]. We identified codes related to services and service bundles for methadone treatment consistent with the components of Medicare’s OTP weekly service bundle [13].

Using this initial broad list of codes, we examined the availability of fee-for-service fees for each code in every state (and the District of Columbia) to identify a subset of codes paid for in most states. We also assessed the frequency of claims for these codes in Medicaid encounter and fee-for-service claims from multiple quarters between 2014 and 2016, and analysis of more current claims data (e.g. 2019 to 2021) for three Medicaid programs in Virginia, Kentucky, and Maryland through partnerships with state-based researchers to assist in selecting key services and codes that are most relevant to OUD treatment.

Through this process, we identified a consistent set of codes related to different categories of OUD treatment. First, a regular methadone treatment bundle, includes combinations of approximately 15 codes^1^ that differ across state Medicaid programs with codes related to the following services: methadone treatment bundle fee, methadone dispensing or administration, individual therapy (30 minutes), group therapy (30 minutes), and toxicology testing. This can be thought of as a typical methadone treatment bundle. Second, in several states, Medicaid programs paid for additional services beyond the regular methadone treatment bundle, with codes related to additional care coordination, case management, Opioid Health Home or Center Of Excellence program payments. Third, we identified fifteen commonly billed procedure codes related to four other types of services related to OUD treatment types: evaluation and management, psychotherapy, toxicology testing, and substance use screening and psychiatric diagnostic evaluation.

We collected Medicaid fees from state websites in March 2021 for the selected service codes Table 1 and Appendix Table 1**)**. To limit payment differences due to provider type and setting, we collected data for a selection of providers such as physicians and psychiatrists (excluding mid-level providers when identified) in outpatient settings. We included analogous individual services when a state methadone bundle did not contain the full range of services included in the Medicare bundle. We identified Medicare fees using the Physician Fee Schedule Look-Up Tool [19] and calculated the relevant Medicare fees using relative value units, geographic practice cost indexes, and conversion factors from CMS [20, 21]. Summary statistics of the average Medicaid and Medicare fees for each procedure code across states are shown in Appendix Tables 2, 3, and 4, along with the procedure weights for the group of services related to OUD treatment.

**Table 1:** Reported or estimated weekly Medicaid OTP methadone bundle fee by state, as of March 2021

State	Regular Methadone Bundle Fee Components (weekly)						Additional Payments (weekly)		
	Methadone treatment bundle fee	Dispensing and/or Administration	Individual Therapy (30 minutes)	Group Therapy (30 minutes)	Toxicology Testing	Total Regular Methadone Bundle Fee (weekly)	Care Coordination or Case Management (1 hour except as noted)	Opioid Health Home or Center of Excellence (weekly)	Total Regular Methadone Bundle Fee and Additional Payments (weekly)
Alabama	$119.00	X	X	X	X	$119.00	n.d.	n.d.	$119.00
Alaska	$143.85	X	$63.98	$25.59	$12.60	$246.02	$98.80	n.d.	$344.82
Arizona^a^	$26.32	X	$40.50	$13.94	$12.60	$93.36	$27.22	n.d.	$202.24
Arkansas	n.d.	n.d.	n.d.	n.d.	n.d.	-—	n.d.	n.d.	-—
California	$99.40	X	X	X	X	$99.40	n.d.	n.d.	$99.40
Colorado^a^	$103.81	X	$45.54	$15.08	$12.89	$177.32	$34.20	n.d.	$211.52
Connecticut	$92.69	X	X	X	X	$92.69	n.d.	n.d.	$92.69
Delaware^a,b^	$28.00	X	$38.66	$6.44	$8.20	$81.30	n.d.	n.d.	$81.30
District of Columbia^a^	$60.06	X	$57.62	$7.21	$8.80	$133.69	$105.68	n.d.	$239.37
Florida	$67.48	X	X	X	X	$67.48	n.d.	n.d.	$67.48
Georgia	$121.80	X	X	X	X	$121.80	n.d.	n.d.	$121.80
Hawaii	n.d.	n.d.	n.d.	n.d.	n.d.	-—	n.d.	n.d.	-—
Idaho	n.d.	n.d.	n.d.	n.d.	n.d.	-—	n.d.	n.d.	-—
Illinois^a^	$70.00	X	$31.06	$11.74	$4.49	$117.29	$50.80	n.d.	$168.09
Indiana	$112.35	X	X	X	X	$112.35	n.d.	n.d.	$112.35
Iowa^c^	$199.09	X	X	X	X	$199.09	n.d.	n.d.	$199.09
Kansas	n.d.	n.d.	n.d.	n.d.	n.d.	-—	n.d.	n.d.	-—
Kentucky	$105.00	X	X	X	X	$105.00	n.d.	n.d.	$105.00
Louisiana	$114.31	X	X	X	X	$114.31	n.d.	n.d.	$114.31
Maine	$81.60	X	X	X	X	$81.60	n.d.	$42.40	$124.00
Maryland ^a^	$74.10	X	$46.36	$22.61	X	$143.07	n.d.	$26.45	$169.52
Massachusetts^a^	$78.82	X	$40.22	$11.76	X	$130.80	$78.52	n.d.	$209.32
Michigan^d^	n.d.	n.d.	n.d.	n.d.	n.d.	-—	n.d.	$104.45	$104.45
Minnesota	$93.73	X	X	X	X	$93.73	n.d.	n.d.	$93.73
Mississippi	n.d.	n.d.	n.d.	n.d.	n.d.	-—	n.d.	n.d.	-—
Missouri	$52.78	X	X	X	X	$52.78	n.d.	n.d.	$52.78
Montana	$125.00	X	X	X	X	$125.00	n.d.	n.d.	$125.00
Nebraska^c^	$199.08	X	X	X	X	$199.08	n.d.	n.d.	$199.08
Nevada	$25.97	X	$54.32	$28.06	$13.36	$121.71	$32.40	n.d.	$154.11
New Hampshire	$76.09	X	$69.11	$28.26	$9.32	$182.78	n.d.	n.d.	$182.78
New Jersey	$91.15	X	X	X	X	$91.15	n.d.	n.d.	$91.15
New Mexico	$120.54	X	X	X	X	$120.54	n.d.	n.d.	$120.54
New York^c^	$207.49	X	X	X	X	$207.49	n.d.	n.d.	$207.49
North Carolina^a^	$116.20	X	$39.62	$5.12	$14.29	$175.23	$81.25 (weekly)	n.d.	$256.48
North Dakota^c^	$207.49	X	X	X	X	$207.49	n.d.	n.d.	$207.49
Ohio	$114.66	X	$48.99	$19.27	$6.67	$189.59	$78.16	n.d.	$267.75
Oklahoma	n.d.	n.d.	n.d.	n.d.	n.d.	-—	n.d.	n.d.	-—
Oregon^a^	$34.93	X	$53.02	$46.80	$12.63	$147.38	$90.00	n.d.	$237.62
Pennsylvania^a^	$52.50	X	$50.00	$3.50	$11.97	$117.97	$30.00	$69.31	$217.28
Rhode Island	$87.52	X	X	X	X	$87.52	n.d.	$53.50	$141.02
South Carolina^c^	$102.04	X	X	X	X	$102.04	n.d.	n.d.	$102.04
South Dakota	n.d.	n.d.	n.d.	n.d.	n.d.	-—	n.d.	n.d.	-—
Texas^a^	$77.00	X	$29.00	$28.00	$13.10	$147.10	n.d.	n.d.	$147.10
Utah	$40.95	X	$59.82	$6.96	$5.02	$112.75	$67.32	n.d.	$180.07
Vermont^e^	$105.00	X	X	X	X	$105.00	n.d.	$34.81	$139.81
Virginia^a,f^	$56.00	X	$48.00	$14.50	$14.96	$133.46	$60.75	n.d.	$194.21
Washington	$110.67	X	X	X	X	$110.67	n.d.	n.d.	$110.67
West Virginia	$105.00	X	X	X	X	$105.00	n.d.	n.d.	$105.00
Wisconsin^a,g^	$73.14	X	$54.04	$3.16	$57.66	$188.00	$43.28	n.d.	$231.28
Wyoming	n.d.	n.d.	n.d.	n.d.	n.d.	-—	n.d.	n.d.	-—
Medicare	$212.00	X	X	X	X	$212.00			$212.00

*Source*: Authors' analysis of Medicaid physician fees posted to state websites as of March 2021; Tennessee is excluded because it does not establish fee-for-service Medicaid provider fees

*Notes*: "X" indicates service covered in Medicaid OTP Methadone bundle fee; n.d. = no data, either data not available or service not covered; -— = could not be computed due to lack of data; Medicare 2021 national rate; OHH = Opioid Health Home. No data was available for any relevant payment for Arkansas, Hawaii, Idaho, Kansas, Michigan (except OHH payments), Oklahoma, South Dakota, and Wyoming. Duration varies for H0004 and H0005, so each is converted to a per 30-minute fee for purposes of the estimated weekly bundle fee. Care coordination/case management duration also varies, so each is converted to a per 1 hour fee

The regular methadone bundle fee (weekly) is based on the Medicare HCPCS G2067, "Medication assisted treatment, methadone; weekly bundle including dispensing and/or administration, substance use counseling, individual and group therapy, and toxicology testing, if performed (provision of the services by a Medicare-enrolled Opioid Treatment Program)." We used combinations of the HCPCS and CPT codes H0020, H0016, G2067, H0004, 90832, H0005, 90853, H0003, H0048, 80305, H0006, G9012, T1016, T1017, H0032, 81020, and S9445, as appropriate with state-specific codes and usage, to create bundles based on the Medicare HCPCS G2067

^a^Fee rates were adjusted to be consistent with the bundle as described, e.g. Individual Therapy (15 minutes) was multiplied by two to obtain Individual Therapy (30 minutes)

^b^Delaware is not included in the index computation as private communication from the state indicated that the Medicaid FFS rates are very infrequently used and may be substantially lower than the MMC rates for OTP services, thus are not representative of state payments generally

^c^Uses Medicare G Codes

^d^This rate is the weekly estimate of Michigan's S0280 HG Recovery Action Plan Rate for the first month of OHH care at an OTP; subsequent months have lower payment

^e^Vermont's health home fee is an unweighted average of their fee for hubs and spokes

^f^Virginia reimburses for one month of care coordination, which has been divided by four for a weekly rate

^g^Wisconsin only covers 6 units of service per week, so the daily rate is multiplied by 6 rather than 7 as in other states. Wisconsin also only covers 39 units of H0003 per year, so it is multiplied by 3/4 to get a weekly rate

The Medicaid fee index measures each state’s average Medicaid fee relative to the national average Medicaid fee for the same group of procedures or for the OTP bundle. We estimated average Medicaid fees using a simple average fee across provider types for each procedure code in each state. To estimate national average Medicaid fees for each procedure, we weighted state fees by March 2021 Medicaid enrollment, which reflected the point-in-time number of nonelderly adult Medicaid enrollees not dually enrolled in Medicare in March 2021, estimated using factors computed from MSIS and CMS dual and child enrollment data. We calculated the ratio of average Medicaid fee to the national average Medicaid fee for each procedure in each state. For the index of services related to OUD treatment, we combined the state ratios into a Medicaid fee index and respective sub-indexes for the four subgroups (evaluation and management, psychotherapy, toxicology testing, and substance use screening and diagnostic evaluation) using equal weights for the four subgroups, and equal weights for the codes within each of the four subgroups. This weighting scheme was used because estimates reflecting national expenditure weights across these services proved infeasible to produce.

The Medicaid-to-Medicare fee index measures each state’s average Medicaid fee relative to the average Medicare fee for the same group of procedures or for the OTP bundle, and is calculated similarly to the Medicaid fee index Tables 2, 3. We weighted locality-level Medicare fees by Medicare enrollment to calculate average Medicare fees in states with multiple substate Medicare fee localities. We weighted state indexes by March 2021 Medicaid enrollment for each state to calculate the national average Medicaid-to-Medicare fee index. This weighting provides overall estimates that are more representative of the average patient experience across the country. For additional context, we examined the correlation between the Medicaid-to-Medicare fee index for the regular methadone treatment bundle with: a previously published Medicaid-to-Medicare index based on a broad range of fees in 2019 including twenty-seven common procedures covering primary care, obstetrical care, and other services [5], the share of state Medicaid enrollees treated for OUD in 2019 [7], state Medicaid enrollment in 2021, and the share of Medicaid enrollees who were Black in 2019 (Appendix Table 5). The last correlation is a preliminary inquiry into equity concerns.

This study had several limitations. Medicaid managed care organizations’ fees were excluded since they were largely unavailable for research purposes, and no fees related to Tennessee were collected because this state has no fee-for-service Medicaid fees. According to CMS, 69.6 percent of Medicaid beneficiaries were enrolled in comprehensive managed care in 2018 [22]. A twenty-state survey conducted by the Government Accountability Office found that managed care plans paid fees similar to fee-for-service Medicaid in most states (within 5 percent or less), with some variation among states, and Medicaid managed care payments were generally equal to or higher than Medicaid FFS [23]. Thus, since the study sample excludes managed care fees, results cannot be generalized to the overall Medicaid programs. As a consequence, the Medicaid-to-Medicare fee ratios may be biased, likely downwards, for states with high Medicaid managed care penetration. It is important that the findings of this study are reexamined with data such as CMS’s Medicaid managed care encounter cost data; however, that data is confidential, and access is highly restricted. In addition, we were unable to collect fee codes related to buprenorphine treatment bundles consistently across states, and thus were constrained to study methadone treatment bundles and other related OUD treatment services. Other services related to OUD care, such as residential care and recovery services, were determined to be out of scope and excluded from study.

## Results

Weekly Medicaid fee bundles for methadone treatment at OTPs in 2021, reported in state fee schedules or estimated from state fee schedules based on the components of the Medicare weekly methadone treatment bundle (the regular methadone bundle), were computed for 41 states and DC. These fees varied widely, up to more than 4-fold across states—from $52.78 in Missouri and $67.48 in Florida to $246.02 in Alaska and $207.49 in both New York and North Dakota Table 1. The national average fee for the regular methadone bundle in Medicare was $212.00, with modest geographic adjustments across states (Appendix Table 2). In Medicaid programs, we reviewed state websites and literature to identify additional care coordination or case management fees beyond the regular methadone bundle services we identified for 12 states and DC, and weekly Opioid Health Home or Center of Excellence bundle fees were identified for an additional 6 states. These additional payment components were identified in 18 states and DC. The highest fees including these additional payments were $344.82 in Alaska, $267.75 in Ohio, $256.48 in North Carolina, and $239.37 in the District of Columbia. In almost all cases, states with additional payments were states that already paid higher fees for the regular methadone bundle, except for Arizona and Pennsylvania.

The Medicaid fee index for regular methadone treatment at OTPs in 2021 demonstrates the large variation in fees across states, with even larger variation when the addition payments are considered Table 2. Alaska, the District of Columbia, North Carolina, Ohio and Oregon all have Medicaid fee indexes for the methadone bundle with additional payments that are over 150 percent of the national average, while Missouri and Florida are below 50 percent of the national average.

**Table 2:** Medicaid fee index and Medicaid-to-Medicare fee index, by OTP methadone bundle type and state, as of March 2021

State	2021 Medicaid fee index		2021 Medicaid-to-Medicare fee index
	Regular Methadone Bundle Fee	Regular Methadone Bundle Fee and Additional Payments	Regular Methadone Bundle Fee
US	1.00	1.00	0.56
Alabama	0.92	0.77	0.59
Alaska	1.90	2.23	0.94
Arizona	0.72	1.31	0.45
Arkansas	n.d.	n.d.	n.d.
California	0.77	0.64	0.45
Colorado	1.37	1.37	0.83
Connecticut	0.71	0.60	0.41
Delaware	n.d.	n.d.	n.d.
District of Columbia	1.03	1.55	0.56
Florida	0.52	0.44	0.32
Georgia	0.94	0.79	0.60
Hawaii	n.d.	n.d.	n.d.
Idaho	n.d.	n.d.	n.d.
Illinois	0.90	1.09	0.56
Indiana	0.87	0.73	0.56
Iowa	1.53	1.29	0.99
Kansas	n.d.	n.d.	n.d.
Kentucky	0.81	0.68	0.52
Louisiana	0.88	0.74	0.56
Maine	0.63	0.80	0.40
Maryland	1.10	1.10	0.66
Massachusetts	1.01	1.36	0.59
Michigan	n.d.	n.d.	n.d.
Minnesota	0.72	0.61	0.45
Mississippi	n.d.	n.d.	n.d.
Missouri	0.41	0.34	0.26
Montana	0.96	0.81	0.59
Nebraska	1.53	1.29	1.00
Nevada	0.94	1.00	0.57
New Hampshire	1.41	1.18	0.85
New Jersey	0.70	0.59	0.40
New Mexico	0.93	0.78	0.59
New York	1.60	1.34	0.95
North Carolina	1.35	1.66	0.85
North Dakota	1.60	1.34	1.00
Ohio	1.46	1.73	0.92
Oklahoma	n.d.	n.d.	n.d.
Oregon	1.14	1.54	0.71
Pennsylvania	0.91	1.41	0.57
Rhode Island	0.67	0.91	0.40
South Carolina	0.79	0.66	0.51
South Dakota	n.d.	n.d.	n.d.
Texas	1.13	0.95	0.71
Utah	0.87	1.17	0.55
Vermont	0.81	0.91	0.50
Virginia	1.03	1.26	0.63
Washington	0.85	0.72	0.52
West Virginia	0.81	0.68	0.52
Wisconsin	1.45	1.50	0.93
Wyoming	n.d.	n.d.	n.d.

Sources: Authors' analysis of Medicaid physician fees posted to state websites as of March 2021; Tennessee is excluded because it does not establish fee-for-service Medicaid provider fees

Note: n.d. = no data, either data not available or service not covered; Medicare 2021 national rate; OHH = Opioid Health Home. No data was available for any relevant payment for Arkansas, Hawaii, Idaho, Kansas, Michigan (except OHH payments), Oklahoma, South Dakota, and Wyoming. The additional services in the weekly bundle with additional services are care coordination, health home, and/or center of excellence services. See Table 1 for services included in each bundle type, by state. National average indexes are sums of state indexes, weighted by March 2021 nonelderly adult Medicaid enrollment

The Medicaid-to-Medicare fee index shows that the national average Medicaid fee bundle was 56 percent of Medicare fees for regular methadone treatment at OTPs in 2021. This index varies widely, with almost a 4-fold difference across states. In these estimates, which exclude the additional payment made by many state Medicaid programs to boost services beyond the regular methadone bundle, nine states are below 50 percent of the Medicare fee bundle: Arizona, California, Connecticut, Florida, Maine, Minnesota, Missouri, New Jersey, and Rhode Island.

**Fig. 1:**
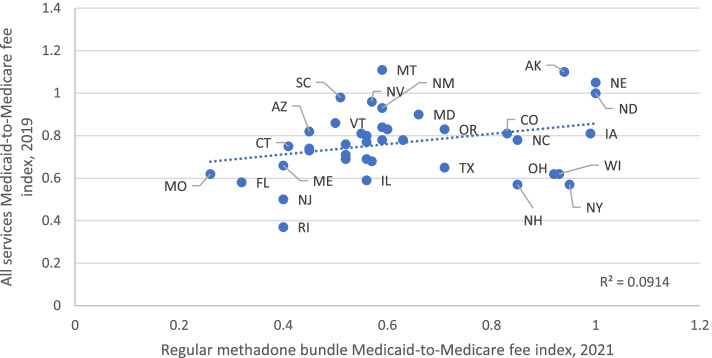
Medicaid-to-Medicare fee index for selected common procedures and the regular methadone bundle, by state

Overall, the state Medicaid-to-Medicare fee index for the regular methadone fee bundle varies positively with an index based on fees from a broad range of twenty-seven common services in 2019 (R^2^ = 0.0914, Fig. 1; rank correlation of 0.35, (Appendix Table 5). Also for the regular methadone bundle fee, states with a higher share of Medicaid enrollees with OUD or a higher Medicaid enrollment in 2019 had a lower Medicaid-to-Medicare fee index than states with lower OUD rates or enrollment (rank correlation of −0.16 and −0.12, respectively). In addition, we find that states with a higher share of Medicaid enrollees who are Black had a lower Medicaid-to-Medicare fee index for the regular methadone fee bundle (rank correlation of −0.05).

We also examined Medicaid fees for four types of services related to OUD treatment by state and related national Medicare fees as of 2021. Across states, Medicaid fees varied widely across the components of each of the four services, with differences commonly 5-fold and larger across states (Appendix Table 1 and Appendix Table 3). For these four services combined, the Medicaid-to-Medicare fee index shows that the national average Medicaid fees for these four services was 64 percent of Medicare fees in 2021 Table 3). The Medicaid-to-Medicare fee index was lower for evaluation & management (0.52) than for alcohol or substance use screening and psychiatric diagnostic evaluation (0.63) or for psychotherapy (0.67), and highest for toxicology testing (0.76).

**Table 3:** Medicaid fee index and Medicaid-to-Medicare fee index for services related to OUD treatment, by service type and state, as of March 2021

**State**	2021 Medicaid fee index					2021 Medicaid-to-Medicare fee index				
	**All services**	Component services				**All services**	Component services			
		**E & M**	**Psychotherapy**	**Toxicology testing**	**Substance use screening and psychiatric evaluation**		**E & M**	**Psychotherapy**	**Toxicology testing**	**Substance use screening and psychiatric evaluation**
US	1.00	1.00	1.00	1.00	1.00	0.64	0.52	0.67	0.76	0.63
Alabama	0.97	0.97	1.09	0.91	0.86	0.65	0.53	0.75	0.71	0.53
Alaska	2.01	2.33	2.28	1.31	2.11	1.02	0.94	1.09	1.00	1.05
Arizona	1.35	1.26	1.46	1.32	1.36	0.88	0.67	0.99	1.01	0.84
Arkansas	1.28	0.96	1.46	1.48	1.32	0.89	0.55	1.05	1.21	0.84
California	0.78	0.55	0.74	0.93	1.01	0.50	0.27	0.47	0.72	0.58
Colorado	1.10	1.37	0.92	0.84	1.18	0.70	0.70	0.61	0.69	0.80
Connecticut	1.03	1.12	1.11	0.73	1.02	0.62	0.54	0.71	0.60	0.59
Delaware	1.57	1.60	1.52	1.67	1.49	1.03	0.82	1.00	1.29	0.99
District of Columbia	1.23	1.46	1.22	1.05	1.19	0.73	0.66	0.74	0.80	0.72
Florida	0.86	0.74	0.94	0.90	0.91	0.56	0.39	0.63	0.73	0.55
Georgia	1.11	0.93	1.21	1.16	1.13	0.76	0.51	0.84	0.89	0.80
Hawaii	0.91	0.80	1.14	0.79	0.91	0.58	0.39	0.75	0.61	0.53
Idaho	1.28	1.47	1.23	1.24	1.10	0.85	0.83	0.86	0.95	0.70
Illinois	0.91	0.68	1.20	0.79	1.07	0.60	0.36	0.82	0.61	0.65
Indiana	1.06	1.09	1.02	1.15	0.98	0.72	0.60	0.71	0.89	0.70
Iowa	1.36	1.00	1.74	1.26	1.43	0.94	0.55	1.23	0.98	1.00
Kansas	0.91	0.93	0.75	1.14	0.90	0.63	0.52	0.53	0.93	0.72
Kentucky	1.04	0.98	0.95	1.31	0.92	0.71	0.54	0.66	1.00	0.65
Louisiana	1.23	1.57	1.03	1.10	1.21	0.81	0.85	0.71	0.85	0.84
Maine	0.80	0.86	0.75	0.92	0.68	0.54	0.47	0.52	0.70	0.49
Maryland	1.40	1.59	1.52	0.99	1.60	0.86	0.79	1.00	0.75	0.94
Massachusetts	1.09	1.20	1.29	0.85	1.01	0.68	0.59	0.84	0.65	0.66
Michigan	0.87	0.88	0.78	1.09	0.74	0.58	0.47	0.53	0.83	0.51
Minnesota	1.26	1.39	1.20	1.31	1.13	0.83	0.73	0.81	1.00	0.77
Mississippi	1.21	1.29	1.20	1.18	1.12	0.81	0.73	0.84	0.90	0.71
Missouri	1.06	1.40	1.16	1.05	0.63	0.71	0.77	0.81	0.80	0.45
Montana	1.66	1.98	1.75	1.31	1.58	1.07	1.02	1.18	1.00	1.06
Nebraska	1.73	1.55	2.20	1.31	2.00	1.15	0.86	1.54	1.00	1.26
Nevada	1.15	0.94	1.18	1.18	1.28	0.76	0.48	0.79	0.91	0.87
New Hampshire	1.17	1.66	0.89	0.83	1.30	0.74	0.85	0.59	0.65	0.88
New Jersey	1.03	1.64	0.79	1.18	0.50	0.63	0.77	0.51	0.90	0.32
New Mexico	1.20	1.38	1.30	1.23	0.87	0.79	0.75	0.89	0.94	0.58
New York	0.70	0.80	0.82	0.30	0.90	0.42	0.40	0.54	0.24	0.53
North Carolina	1.18	1.16	1.13	1.27	1.17	0.80	0.63	0.78	0.98	0.82
North Dakota	1.42	1.60	1.43	1.31	1.35	0.94	0.85	0.97	1.00	0.93
Ohio	1.21	1.73	0.89	1.04	1.19	0.77	0.93	0.61	0.79	0.73
Oklahoma	1.25	1.40	1.29	1.17	1.12	0.84	0.77	0.89	0.89	0.77
Oregon	1.24	1.38	1.61	0.91	1.00	0.81	0.74	1.10	0.70	0.69
Pennsylvania	0.77	0.99	0.42	1.05	0.43	0.50	0.53	0.28	0.82	0.26
Rhode Island	0.83	0.51	0.89	1.00	1.00	0.55	0.26	0.58	0.79	0.59
South Carolina	1.51	1.16	2.04	1.05	1.60	0.99	0.64	1.40	0.79	1.07
South Dakota	1.13	1.03	1.25	1.31	0.74	0.75	0.55	0.87	1.00	0.46
Texas	1.03	0.72	1.07	1.31	0.99	0.70	0.38	0.73	1.00	0.66
Utah	1.02	1.37	1.04	1.01	0.66	0.68	0.74	0.70	0.78	0.49
Vermont	1.10	0.96	1.23	1.22	1.04	0.74	0.50	0.84	1.00	0.72
Virginia	1.07	1.03	1.02	1.32	0.91	0.72	0.54	0.69	1.02	0.61
Washington	0.90	0.85	0.77	1.20	0.80	0.60	0.43	0.51	0.92	0.54
West Virginia	1.10	1.09	1.01	1.31	0.98	0.75	0.60	0.69	1.00	0.68
Wisconsin	1.22	0.92	1.67	1.08	1.18	0.82	0.51	1.17	0.84	0.74
Wyoming	1.33	1.33	1.32	1.30	1.39	0.85	0.69	0.90	1.06	0.84

*Sources*: Authors' analysis of Medicaid physician fees posted to state websites as of March 2021; Tennessee is excluded because it does not establish fee-for-service Medicaid provider fees

*Notes*: E & M = Evaluation and management; Substance use screening and psychiatric evaluation includes alcohol and/or substance use structured screening and brief intervention services and psychiatric diagnostic evaluation with or without medical services. The four component services have equal weights. National average indexes are sums of state indexes, weighted by March 2021 nonelderly adult Medicaid enrollment. See methods section and Appendix Table 1 for more detail about the service weightings and fees

The Medicaid-to-Medicare fee index for all services related to OUD treatment varied widely by state, with a low in New York (0.42) and California and Pennsylvania (0.50) and only four states with fees equal to or higher than Medicare, Alaska (1.02), Delaware (1.03),Montana (1.07), and Nebraska (1.15). See Appendix Table 4 for detail on Medicare’s geographic adjustments. Medicaid fees were low relative to Medicare for almost all state services examined. Only one state had evaluation and management services equal to or higher than Medicare, Montana (1.02). Five states had substance use screening and psychiatric evaluation fees equal to or higher than Medicare, Nebraska (1.26), South Carolina (1.07), Alaska (1.05), Montana (1.06) and Iowa (1.0). Ten states had psychotherapy fees equal to or higher than Medicare, and 15 states had fees equal to or higher than Medicare for toxicology testing.

## Discussion

This research found very wide variation across states in Medicaid fees for OUD treatment services and very low rates relative to Medicare in 2021. Medicaid fee bundles for methadone treatment at OTPs varied widely, up to more than 4-fold across states. This variation across states is similar in the corresponding Medicaid-to-Medicare fee index, which includes geographic adjustments for differences in prices and labor. There appears to be very little justification for such variation, particularly since the services provided in an OTP setting are highly regulated. We found 18 states and DC had Medicaid payments to boost the regular Medicaid methadone fee bundles for such services as additional care coordination or case management fees and Opioid Health Home services, and in most cases, states arranging for additional payments were states that already paid higher fees for the regular methadone bundle. The Medicaid-to-Medicare fee index shows that the national average Medicaid fee bundle was 56 percent of Medicare fees for regular methadone treatment at OTPs in 2021. This is substantially lower than the Medicaid-to-Medicare fee index for other services, which averaged 72 percent across 27 common procedures across a broad range of care, and lower than the 67 percent found for primary care, the lowest fee index across service types [5]. For services related to OUD treatment, Medicaid fees varied up to 5-fold and larger across the components of each of the four services, and Medicaid fees were low relative to Medicare for almost all state services examined. The Medicaid-to-Medicare fee index was 64 percent of Medicare fees in 2021, ranging from 52 percent for evaluation & management to 76 percent for toxicology testing.

In 2021 states with higher rates of Medicaid enrollees treated for OUD, higher Medicaid enrollment and higher shares of Black enrollment had lower Medicaid-to-Medicare fee indexes for methadone bundles than lower-enrollment states, raising questions about how these fees are set and the need for close study of the need for reform in order to achieve the Biden administration’s goals related to increasing access to substance use treatment, and in equity [24]. As low Medicaid fees have been shown to be correlated to lower provider participation in Medicaid and thus imply less access to care for Medicaid enrollees, these findings add to concerns about equitable access to life-saving care during the opioid overdose crisis.

The low Medicaid fees for OUD treatment services across most states persist despite recent efforts to increase access to opioid use disorder treatment for Medicaid enrollees, including the 31 states that increased Medicaid fees for substance use disorder treatment between 2014 and 2019 [11]. The persistently low rates have important implications, as they may limit access to life-saving treatment during the current opioid overdose crisis.

The COVID-19 pandemic drove federal and state changes in services related to OUD treatment in Medicaid and Medicare, including that Medicare as well as Medicaid programs in 42 states and the District of Columbia implemented payment parity for at least some telehealth services compared to face-to-face services by January 2021 [25]. Thus the data collected for this study likely applies to a more flexible set of services that originally intended when payment rates were set. It is not clear whether payment parity will continue after the public health emergency ends, and some have speculated that telehealth visits may be covered at a lower rate than during the COVID-19 pandemic [26]. Further, early evidence suggests that the pandemic related flexibilities added to federal- and state-level policies may have steadied OUD treatment, particularly for buprenorphine while some decreases were seen in treatment initiation, urine screens and OTP services, at least initially [27–29].

Future research can aim to better understand how variation in payment rates in these data may explain variation in access to OUD treatment services is necessary to explore policy options to address shortcomings in access and equity. For example, researchers can examine the association between fees and patient access or provider participation. Future research can also assess the extent to which states with low Medicaid fees for OUD treatment services have low Medicaid fees for other types of care.

## Conclusions

In this study, we present the first examination of Medicaid professional fees related to the treatment of OUD to assess how Medicaid fees compared with Medicare fees in 2021 across states and services. We find that Medicaid professional fees for treatment of OUD varied widely across states and were substantially below fees paid by Medicare. In recent years, several states increased these Medicaid fees, in part, attempts to address long-standing concerns that low Medicaid physician fees for effective or recommended services may impede access to care for enrollees with OUD. This study suggests that these concerns are still relevant and that more must be done to facilitate access to life-saving treatment for Medicaid enrollees, an urgent priority in the face of the current opioid overdose crisis.

## Supplementary Information

**Additional file 1.**

## Data Availability

The data collected and analyzed for this study has been made publicly available by the authors. Clemans-Cope L. Medicaid and Medicare Fees and Indexes for a Weekly Opioid Treatment Program Methadone Bundle and Selected Other Services Related to the Treatment of Opioid Use Disorder [Internet]. Urban Data Catalog. 2021 [cited 2021 Dec 22]. Available from: https://datacatalog.urban.org/dataset/medicaid-and-medicare-fees-and-indexes-weekly-opioid-treatment-program-methadone-bundle-and

## Footnotes

1 We used combinations of the HCPCS and CPT codes H0020, H0016, G2067, H0004, 90832, H0005, 90853, H0003, H0048, 80305, H0006, G9012, T1016, T1017, H0032, 81020, and S9445, as appropriate with state-specific codes and usage, to create bundles based on the Medicare HCPCS G2067.

## References

[1] Friedman J, Akre S (2021). COVID-19 and the Drug Overdose Crisis: Uncovering the Deadliest Months in the United States, January–July 2020. Am J Public Health..

[2] Mann B. Drug Overdose Deaths Spiked To 88,000 During The Pandemic, White House Says [Internet]. NPR.org. 2021 [cited 2021 May 14]. Available from: https://www.npr.org/2021/04/01/983414684/white-house-says-drug-overdose-deaths-spiked-to-88-000-during-the-pandemic. Accessed 14 May 2021.

[3] Wakeman SE, Larochelle MR, Ameli O, Chaisson CE, McPheeters JT, Crown WH (2020). Comparative Effectiveness of Different Treatment Pathways for Opioid Use Disorder. JAMA Netw Open..

[4] Donohue J, Raslevich AC, Cole E (2020). Medicaid’s Role in Improving Substance Use Disorder Treatment in the US [Internet].

[5] Zuckerman S, Skopec L, Aarons J (2021). Medicaid Physician Fees Remained Substantially Below Fees Paid By Medicare In 2019. Health Affairs..

[6] Clemans-Cope L, Winiski E, Lynch V, Epstein M. Rapid Growth in Medicaid Prescriptions and Spending to Treat Opioid Use Disorder and Opioid Overdose from 2010 to 2018 [Internet]. Urban Institute. 2020 [cited 2020 Oct 5]. Available from: https://www.urban.org/research/publication/rapid-growth-medicaid-prescriptions-and-spending-treat-opioid-use-disorder-and-opioid-overdose-2010-2018. Accessed 5 Oct 2020.

[7] US DHHS. Report to Congress: T-MSIS Substance Use Disorder (SUD) Data Book, Treatment of SUD in Medicaid, 2018. 2021; Available from: https://www.medicaid.gov/medicaid/data-systems/downloads/2018-sud-data-book.pdf

[8] CMS. CMS Issues Guidance about Expanded Medicaid Coverage for Treatment of Opioid Use Disorders [Internet]. Centers for Medicare & Medicaid Services (CMS). 2020 [cited 2021 Oct 6]. Available from: https://www.cms.gov/newsroom/news-alert/cms-issues-guidance-about-expanded-medicaid-coverage-treatment-opioid-use-disorders. Accessed 6 Oct 2021.

[9] Clemans-Cope L, Wishner JB, Allen EH, Lallemand N, Epstein M, Spillman BC (2017). Experiences of three states implementing the Medicaid health home model to address opioid use disorder—Case studies in Maryland, Rhode Island, and Vermont. J Substance Abuse Treatment..

[10] Ghertner R (2019). U.S. trends in the supply of providers with a waiver to prescribe buprenorphine for opioid use disorder in 2016 and 2018. Drug Alcohol Depend..

[11] GAO. Medicaid: States’ Changes to Payment Rates for Substance Use Disorder Services. U S Government Accountability Office (GAO) [Internet]. 2020 30 [cited 2020 Feb 5];(GAO-20-260). Available from: https://www.gao.gov/products/GAO-20-260. Accessed 5 Feb 2020.

[12] Holgash K, Heberlein M. Physician Acceptance Of New Medicaid Patients: What Matters And What Doesn’t [Internet]. Health Affairs Blog. 2019 [cited 2021 Oct 6]. Available from: https://www.healthaffairs.org/do/10.1377/hblog20190401.678690/full/. Accessed 6 Oct 2021.

[13] CMS. Medicare Enrollment of Opioid Treatment Programs and Enhancements to Provider Enrollment Regulations Concerning Improper Prescribing and Patient Harm [Internet]. Federal Register. 2019 [cited 2021 Oct 7]. Available from: https://www.federalregister.gov/documents/2019/11/15/2019-24086/medicare-program-cy-2020-revisions-to-payment-policies-under-the-physician-fee-schedule-and-other. Accessed 7 Oct 2021.

[14] Clemans-Cope L. Medicaid and Medicare Fees and Indexes for a Weekly Opioid Treatment Program Methadone Bundle and Selected Other Services Related to the Treatment of Opioid Use Disorder [Internet]. Urban Data Catalog. 2021; [cited 2021 Dec 22]. Available from: https://datacatalog.urban.org/dataset/medicaid-and-medicare-fees-and-indexes-weekly-opioid-treatment-program-methadone-bundle-and. Accessed 22 Dec 2021.

[15] Shoff C, Yang TC, Shaw BA (2021). Trends in Opioid Use Disorder Among Older Adults: Analyzing Medicare Data, 2013–2018. American Journal of Preventive Medicine..

[16] HHS OIG. Many Medicare Beneficiaries Are Not Receiving Medication to Treat Their Opioid Use Disorder. 2021 Dec;38.

[17] Federal Register. Federal Register :: Medicare Program; CY 2020 Revisions to Payment Policies Under the Physician Fee Schedule and Other Changes to Part B Payment Policies; Medicare Shared Savings Program Requirements; Medicaid Promoting Interoperability Program Requirements for Eligible Professionals; Establishment of an Ambulance Data Collection System; Updates to the Quality Payment Program; Medicare Enrollment of Opioid Treatment Programs and Enhancements to Provider Enrollment Regulations Concerning Improper Prescribing and Patient Harm; and Amendments to Physician Self-Referral Law Advisory Opinion Regulations Final Rule; and Coding and Payment for Evaluation and Management, Observation and Provision of Self-Administered Esketamine Interim Final Rule [Internet]. 2019 [cited 2022 Apr 14]. Available from: https://www.federalregister.gov/documents/2019/11/15/2019-24086/medicare-program-cy-2020-revisions-to-payment-policies-under-the-physician-fee-schedule-and-other. Accessed 14 Apr 2022.

[18] Lynch V, Clemans-Cope L, Wissoker D, Johnson P, Epstein M, Winiski E. Behavioral Health Services Algorithm. Version 3. Urban Institute. 2021.

[19] CMS. Medicare: Physician Fee Schedule, Physician Fee Schedule Look-Up Tool [Internet]. U.S. Centers for Medicare & Medicaid. 2021 [cited 2021 Oct 7]. Available from: https://www.cms.gov/Medicare/Medicare-Fee-for-Service-Payment/PFSlookup. Accessed 7 Oct 2021.

[20] CMS. Medicare: Clinical Laboratory Fee Schedule, Clinical Laboratory Fee Schedule Files [Internet]. U.S. Centers for Medicare & Medicaid. 2016 [cited 2021 Oct 7]. Available from: https://www.cms.gov/Medicare/Medicare-Fee-for-Service-Payment/ClinicalLabFeeSched/Clinical-Laboratory-Fee-Schedule-Files. Accessed 7 Oct 2021.

[21] CMS. Medicare: Physician Fee Schedule, PFS Relative Value Files [Internet]. U.S. Centers for Medicare & Medicaid. 2019 [cited 2021 Oct 7]. Available from: https://www.cms.gov/Medicare/Medicare-Fee-for-Service-Payment/PhysicianFeeSched/PFS-Relative-Value-Files. Accessed 7 Oct 2021.

[22] Mathematica, CMS. Medicaid Managed Care Enrollment and Program Characteristics, 2018 [Internet]. 2020 [cited 2021. Available from: https://www.medicaid.gov/medicaid/managed-care/downloads/2018-medicaid-managed-care-enrollment-report.pdf. Accessed 7 Oct 2021.

[23] GAO. Medicaid Payment: Comparisons of Selected Services under Fee-for-Service, Managed Care, and Private Insurance. 2014 [cited 2017 Jul 17];(GAO-14-533). Available from: http://www.gao.gov/products/GAO-14-533. Accessed 17 July 2017.

[24] ONDCP. The Biden-Harris Administration’s Statement of Drug Policy Priorities for Year One [Internet]. 2021 [cited 2021 Apr 9]. Available from: https://www.whitehouse.gov/wp-content/uploads/2021/03/BidenHarris-Statement-of-Drug-Policy-Priorities-April-1.pdf. Accessed 5 May 2021.

[25] Pessar SC, Boustead A, Ge Y, Smart R, Pacula RL (2021). Assessment of State and Federal Health Policies for Opioid Use Disorder Treatment During the COVID-19 Pandemic and Beyond. JAMA Health Forum..

[26] Ellimoottil C. Understanding The Case For Telehealth Payment Parity [Internet]. Health Affairs Forefront. 2021 [cited 2022 Apr 26]. Available from: https://www.healthaffairs.org/do/10.1377/forefront.20210503.625394/full/

[27] Cantor J, Dick AW, Haffajee R, Pera MF, Bravata DM, Stein BD (2021). Use of buprenorphine for those with employer-sponsored insurance during the initial phase of the COVID-19 pandemic. J Subst Abuse Treat..

[28] Huskamp HA, Busch AB, Uscher-Pines L, Barnett ML, Riedel L, Mehrotra A (2020). Treatment of Opioid Use Disorder Among Commercially Insured Patients in the Context of the COVID-19 Pandemic. JAMA..

[29] Tilhou AS, Dague L, Saloner B, Beemon D, Burns M (2022). Trends in Engagement With Opioid Use Disorder Treatment Among Medicaid Beneficiaries During the COVID-19 Pandemic. JAMA Health Forum..

