# Supplementary material for: Medicaid professional fees for treatment of opioid use disorder varied widely across states and were substantially below fees paid by medicare in 2021
Source: Subst Abuse Treat Prev Policy. 2022 Jul 6;17:49. doi: 10.1186/s13011-022-00478-y (PMC9257570; doi:10.1186/s13011-022-00478-y)
Supplement: Supplementary file 1 — Additional file 1. [file 13011_2022_478_MOESM1_ESM.docx]

# **Appendix Table 1. Medicaid Fees for Other Services Related to OUD Treatment (Evaluation & Management, Psychotherapy, Toxicology Testing, Psychiatric Diagnostic Evaluation & Alcohol/Substance Use Screening) by State, Based on the Medicare OTP Methadone Bundle Fee Components, as of March 2021, and National Medicare Fees as of 2021**

|  | Evaluation and Management (E&M) | | | Psychotherapy | | |
| --- | --- | --- | --- | --- | --- | --- |
|  | Ongoing evaluation and management, straightforward complexity | Ongoing evaluation and management, low complexity | Ongoing evaluation and management, medium complexity | Psychotherapy, 30 minutes with patient and/or family member | Psychotherapy, 45 minutes with patient and/or family member | Group psychotherapy (other than multifamily) |
| States Reporting | 50 | 50 | 50 | 50 | 50 | 50 |
| States Covering | 50 | 50 | 50 | 50 | 50 | 50 |
| Alabama | $31.00 | $42.00 | $67.00 | $49.93 | $66.38 | $25.05 |
| Alaska | $66.09 | $111.82 | $163.76 | $114.11 | $151.84 | $45.19 |
| Arizona | $36.30 | $60.30 | $87.52 | $72.87 | $97.29 | $28.92 |
| Arkansas | $27.65 | $42.73 | $71.43 | $46.38 | $69.57 | $47.76 |
| California | $18.10 | $24.00 | $37.50 | $52.87 | $67.16 | $3.47 |
| Colorado | $39.00 | $65.15 | $96.14 | $54.80 | $70.54 | $12.12 |
| Connecticut | $32.58 | $52.15 | $78.94 | $47.67 | $69.88 | $26.45 |
| Delaware | $53.60 | $68.45 | $106.36 | $83.06 | $108.07 | $25.36 |
| District of Columbia | $42.82 | $69.70 | $100.61 | $62.11 | $82.58 | $23.71 |
| Florida | $25.58 | $31.17 | $48.57 | $49.04 | $65.23 | $17.42 |
| Georgia | $29.67 | $40.70 | $62.71 | $53.22 | $68.44 | $30.74 |
| Hawaii | $24.13 | $36.31 | $56.46 | $46.62 | $75.00 | $27.20 |
| Idaho | $43.35 | $70.13 | $100.03 | $61.48 | $81.77 | $24.38 |
| Illinois | $24.25 | $28.35 | $42.50 | $29.48 | $88.40 | $33.70 |
| Indiana | $30.90 | $51.99 | $76.88 | $50.65 | $67.18 | $20.61 |
| Iowa | $29.01 | $51.01 | $64.03 | $53.58 | $101.77 | $51.99 |
| Kansas | $29.76 | $40.84 | $64.22 | $25.45 | $41.54 | $22.24 |
| Kentucky | $31.08 | $42.63 | $67.10 | $47.41 | $62.90 | $19.29 |
| Louisiana | $46.39 | $72.76 | $109.77 | $47.65 | $69.76 | $22.05 |
| Maine | $24.14 | $40.51 | $61.05 | $30.23 | $39.20 | $20.88 |
| Maryland | $46.29 | $75.86 | $109.76 | $55.37 | $105.62 | $37.25 |
| Massachusetts | $34.35 | $57.26 | $84.21 | $52.12 | $86.78 | $30.31 |
| Michigan | $25.36 | $41.80 | $60.62 | $39.03 | $51.90 | $15.45 |
| Minnesota | $41.04 | $66.22 | $94.17 | $62.83 | $82.73 | $21.89 |

**Appendix Table 1 (continued). Medicaid Fees for Other Services Related to OUD Treatment (Evaluation & Management, Psychotherapy, Toxicology Testing, Psychiatric Diagnostic Evaluation & Alcohol/Substance Use Screening) by State, Based on the Medicare OTP Methadone Bundle Fee Components, as of March 2021, and National Medicare Fees as of 2021**

|  | Evaluation and Management (E&M) | | | Psychotherapy | | |
| --- | --- | --- | --- | --- | --- | --- |
|  | Ongoing evaluation and management, straightforward complexity | Ongoing evaluation and management, low complexity | Ongoing evaluation and management, medium complexity | Psychotherapy, 30 minutes with patient and/or family member | Psychotherapy, 45 minutes with patient and/or family member | Group psychotherapy (other than multifamily) |
| States Reporting | 50 | 50 | 50 | 50 | 50 | 50 |
| States Covering | 50 | 50 | 50 | 50 | 50 | 50 |
| Mississippi | $36.95 | $61.64 | $89.83 | $60.07 | $79.90 | $23.81 |
| Missouri | $41.13 | $61.68 | $102.82 | $52.13 | $85.02 | $23.50 |
| Montana | $57.31 | $94.42 | $136.86 | $87.97 | $116.99 | $34.77 |
| Nebraska | $52.25 | $69.71 | $96.05 | $105.00 | $157.50 | $42.66 |
| Nevada | $29.79 | $41.36 | $64.51 | $54.32 | $69.49 | $28.06 |
| New Hampshire | $47.61 | $79.26 | $116.85 | $48.00 | $77.03 | $11.32 |
| New Jersey | $47.69 | $78.00 | $112.82 | $24.50 | $49.00 | $23.00 |
| New Mexico | $39.59 | $65.66 | $96.31 | $61.12 | $85.27 | $28.15 |
| New York | $23.48 | $37.41 | $56.18 | $38.64 | $49.46 | $18.67 |
| North Carolina | $32.83 | $54.82 | $82.60 | $52.24 | $67.85 | $26.58 |
| North Dakota | $46.22 | $76.54 | $110.93 | $71.73 | $95.40 | $28.47 |
| Ohio | $48.97 | $82.85 | $122.27 | $42.99 | $55.89 | $19.27 |
| Oklahoma | $40.14 | $66.86 | $97.32 | $64.72 | $86.09 | $25.61 |
| Oregon | $40.77 | $65.77 | $93.71 | $73.29 | $102.03 | $36.54 |
| Pennsylvania | $40.00 | $40.00 | $54.42 | $26.00 | $39.00 | $3.50 |
| Rhode Island | $20.64 | $20.64 | $27.00 | $37.98 | $80.00 | $14.40 |
| South Carolina | $33.34 | $55.27 | $81.15 | $104.94 | $209.88 | $18.88 |
| South Dakota | $32.47 | $45.40 | $71.15 | $44.98 | $74.96 | $34.46 |
| Texas | $22.14 | $33.95 | $47.68 | $50.79 | $67.63 | $23.52 |
| Utah | $39.34 | $65.28 | $94.89 | $59.82 | $106.77 | $6.96 |
| Vermont | $23.34 | $47.75 | $73.71 | $49.71 | $81.89 | $29.11 |
| Virginia | $29.48 | $49.38 | $72.63 | $50.86 | $67.61 | $20.46 |
| Washington | $24.56 | $40.38 | $58.42 | $40.00 | $53.20 | $14.22 |

**Appendix Table 1 (continued). Medicaid Fees for Other Services Related to OUD Treatment (Evaluation & Management, Psychotherapy, Toxicology Testing, Psychiatric Diagnostic Evaluation & Alcohol/Substance Use Screening) by State, Based on the Medicare OTP Methadone Bundle Fee Components, as of March 2021, and National Medicare Fees as of 2021**

|  | Evaluation and Management (E&M) | | | Psychotherapy | | |
| --- | --- | --- | --- | --- | --- | --- |
|  | Ongoing evaluation and management, straightforward complexity | Ongoing evaluation and management, low complexity | Ongoing evaluation and management, medium complexity | Psychotherapy, 30 minutes with patient and/or family member | Psychotherapy, 45 minutes with patient and/or family member | Group psychotherapy (other than multifamily) |
| States Reporting | 50 | 50 | 50 | 50 | 50 | 50 |
| States Covering | 50 | 50 | 50 | 50 | 50 | 50 |
| West Virginia | $31.25 | $52.35 | $76.12 | $50.48 | $67.31 | $20.03 |
| Wisconsin | $29.28 | $40.40 | $63.53 | $62.89 | $83.56 | $49.53 |
| Wyoming | $38.45 | $64.33 | $91.28 | $55.70 | $87.33 | $30.90 |
| Medicare | $56.88 | $92.47 | $131.20 | $77.81 | $103.28 | $27.57 |

**Appendix Table 1 (continued). Medicaid Fees for Other Services Related to OUD Treatment (Evaluation & Management, Psychotherapy, Toxicology Testing, Psychiatric Diagnostic Evaluation & Alcohol/Substance Use Screening) by State, Based on the Medicare OTP Methadone Bundle Fee Components, as of March 2021, and National Medicare Fees as of 2021**

|  | Toxicology Testing | | | | | Alcohol/Substance Use Screening & Psychiatric Diagnostic Evaluation | | | |
| --- | --- | --- | --- | --- | --- | --- | --- | --- | --- |
|  | Testing for presence of drug, read by direct observation | Testing for presence of drug, read by instrument assisted observation | Testing for presence of drug, by chemistry analyzers | Drug test, definitive 1-7 classes | Drug test, definitive 8-14 classes | Alcohol and/or substance abuse structured SBI services; 15 to 30 minutes | Alcohol and/or substance abuse structured SBI services; > 30 minutes | Psychiatric diagnostic evaluation | Psychiatric diagnostic evaluation with medical services |
| States Reporting | 50 | 49 | 50 | 43 | 40 | 31 | 28 | 49 | 49 |
| States Covering | 50 | 49 | 50 | 43 | 40 | 31 | 28 | 49 | 49 |
| Alabama | $10.47 | $13.37 | $55.87 | $55.48 | $86.09 |  |  | $106.83 | $89.85 |
| Alaska | $12.60 | $17.14 | $62.14 | $114.43 | $156.59 | $56.71 | $110.45 | $232.59 | $256.51 |
| Arizona | $12.60 | $17.14 | $64.65 | $114.43 | $156.59 |  |  | $149.51 | $165.28 |
| Arkansas | $14.96 | $19.95 | $79.81 |  |  |  |  | $155.80 | $148.18 |
| California | $10.08 | $13.71 | $49.71 | $63.95 | $98.39 |  |  | $128.08 | $103.25 |
| Colorado | $12.47 | $13.94 | $15.78 |  |  | $31.96 | $65.41 | $108.61 | $157.47 |
| Connecticut | $7.55 | $10.07 | $38.00 |  |  |  |  | $114.32 | $122.16 |
| Delaware | $18.85 | $25.64 | $92.96 | $108.60 | $160.85 | $37.60 | $72.90 | $190.68 | $172.37 |
| District of Columbia | $10.08 | $13.71 | $49.71 | $91.54 | $125.27 | $32.82 | $63.54 | $127.38 | $141.30 |
| Florida | $9.03 | $12.04 | $48.15 |  |  |  |  | $100.33 | $111.03 |
| Georgia | $11.47 | $15.29 | $61.16 | $94.12 | $128.79 | $33.41 | $65.51 | $128.24 | $105.37 |
| Hawaii | $8.98 | $11.97 | $47.89 | $47.96 | $73.79 |  |  | $104.43 | $104.43 |
| Idaho | $12.11 | $16.16 | $64.65 | $102.99 | $140.93 |  |  | $125.68 | $128.66 |
| Illinois | $8.98 | $11.97 | $47.89 | $47.96 | $73.79 |  |  | $122.11 | $124.44 |
| Indiana | $12.60 | $17.14 | $62.14 | $77.12 | $118.65 | $27.26 | $53.20 | $104.56 | $112.78 |
| Iowa | $14.35 | $19.14 | $76.58 | $75.94 | $116.84 | $33.78 | $65.95 | $171.34 | $197.70 |
| Kansas | $11.49 | $15.33 | $61.29 |  |  | $24.00 | $48.00 |  |  |
| Kentucky | $12.60 | $17.14 | $62.14 | $114.43 | $156.59 | $20.98 | $53.20 | $104.20 | $115.13 |
| Louisiana | $11.22 | $14.96 | $59.86 | $85.82 | $117.44 | $47.65 |  | $108.39 | $108.39 |
| Maine | $8.41 | $11.22 | $44.89 | $82.36 | $112.69 | $21.04 | $41.08 | $72.58 | $60.42 |
| Maryland | $10.02 | $10.02 | $49.40 | $90.97 | $124.49 |  |  | $184.32 | $184.32 |
| Massachusetts | $8.92 | $11.89 | $47.55 | $59.69 | $91.84 | $27.10 | $52.72 | $121.68 | $110.83 |

**Appendix Table 1 (continued). Medicaid Fees for Other Services Related to OUD Treatment (Evaluation & Management, Psychotherapy, Toxicology Testing, Psychiatric Diagnostic Evaluation & Alcohol/Substance Use Screening) by State, Based on the Medicare OTP Methadone Bundle Fee Components, as of March 2021, and National Medicare Fees as of 2021**

|  | Toxicology Testing | | | | | Alcohol/Substance Use Screening & Psychiatric Diagnostic Evaluation | | | |
| --- | --- | --- | --- | --- | --- | --- | --- | --- | --- |
|  | Testing for presence of drug, read by direct observation | Testing for presence of drug, read by instrument assisted observation | Testing for presence of drug, by chemistry analyzers | Drug test, definitive 1-7 classes | Drug test, definitive 8-14 classes | Alcohol and/or substance abuse structured SBI services; 15 to 30 minutes | Alcohol and/or substance abuse structured SBI services; > 30 minutes | Psychiatric diagnostic evaluation | Psychiatric diagnostic evaluation with medical services |
| States Reporting | 50 | 49 | 50 | 43 | 40 | 31 | 28 | 49 | 49 |
| States Covering | 50 | 49 | 50 | 43 | 40 | 31 | 28 | 49 | 49 |
| Michigan | $10.43 | $14.20 | $51.46 | $94.75 | $129.66 | $20.21 | $39.22 | $79.83 | $88.35 |
| Minnesota | $12.60 | $17.14 | $62.14 | $114.43 | $156.59 | $25.43 | $48.59 | $145.27 | $162.61 |
| Mississippi | $11.34 | $15.43 | $55.93 | $102.99 | $140.93 |  |  | $122.74 | $135.68 |
| Missouri | $10.08 | $13.71 | $49.71 | $91.54 | $125.27 | $23.20 | $36.92 | $48.55 | $61.68 |
| Montana | $12.60 | $17.14 | $62.14 | $114.43 | $156.58 | $40.89 | $79.53 | $179.79 | $198.82 |
| Nebraska | $12.60 | $17.14 | $62.14 | $114.43 | $156.59 |  |  | $193.95 | $271.23 |
| Nevada | $13.36 | $17.82 | $71.27 | $71.39 | $109.83 | $41.13 | $80.10 | $131.10 | $106.94 |
| New Hampshire | $9.32 | $12.64 | $50.54 | $50.98 | $78.44 | $39.68 | $76.15 | $131.65 | $131.65 |
| New Jersey | $10.08 | $21.58 | $49.71 | $94.12 | $128.79 | $15.21 | $29.84 | $49.00 | $49.00 |
| New Mexico | $11.84 | $16.11 | $58.41 | $107.56 | $147.19 | $15.56 | $30.86 | $125.05 | $140.56 |
| New York | $5.00 | $5.00 | $7.50 | $15.00 |  |  |  | $93.26 | $115.07 |
| North Carolina | $14.29 | $19.44 | $76.26 | $76.39 | $117.52 | $35.92 | $69.72 | $125.39 | $104.58 |
| North Dakota | $12.60 | $17.14 | $62.14 | $114.43 | $156.59 | $36.61 | $70.99 | $146.79 | $162.69 |
| Ohio | $10.10 | $13.47 | $53.87 | $85.82 | $117.44 |  |  | $130.72 | $144.35 |
| Oklahoma | $11.21 | $15.25 | $55.27 | $101.78 | $139.28 | $33.01 |  | $117.47 | $131.01 |
| Oregon | $8.82 | $12.00 | $43.50 | $80.10 |  | $25.57 | $49.08 | $99.25 | $146.58 |
| Pennsylvania | $11.97 | $15.96 | $63.85 | $63.95 | $98.39 |  |  | $26.25 | $75.00 |
| Rhode Island | $12.51 | $16.68 | $66.73 | $47.96 | $73.79 |  |  | $110.00 | $120.00 |
| South Carolina | $9.83 |  | $50.43 | $89.26 |  | $24.67 | $47.70 | $96.52 | $445.60 |
| South Dakota | $12.60 | $17.14 | $62.14 | $114.43 | $156.59 |  |  | $70.32 | $102.64 |
| Texas | $12.60 | $17.14 | $62.14 | $114.43 | $156.59 | $27.00 |  | $113.91 | $116.81 |

**Appendix Table 1 (continued). Medicaid Fees for Other Services Related to OUD Treatment (Evaluation & Management, Psychotherapy, Toxicology Testing, Psychiatric Diagnostic Evaluation & Alcohol/Substance Use Screening) by State, Based on the Medicare OTP Methadone Bundle Fee Components, as of March 2021, and National Medicare Fees as of 2021**

|  | Toxicology Testing | | | | | Alcohol/Substance Use Screening & Psychiatric Diagnostic Evaluation | | | |
| --- | --- | --- | --- | --- | --- | --- | --- | --- | --- |
|  | Testing for presence of drug, read by direct observation | Testing for presence of drug, read by instrument assisted observation | Testing for presence of drug, by chemistry analyzers | Drug test, definitive 1-7 classes | Drug test, definitive 8-14 classes | Alcohol and/or substance abuse structured SBI services; 15 to 30 minutes | Alcohol and/or substance abuse structured SBI services; > 30 minutes | Psychiatric diagnostic evaluation | Psychiatric diagnostic evaluation with medical services |
| States Reporting | 50 | 49 | 50 | 43 | 40 | 31 | 28 | 49 | 49 |
| States Covering | 50 | 49 | 50 | 43 | 40 | 31 | 28 | 49 | 49 |
| Utah | $11.99 | $15.99 | $51.50 | $64.51 | $99.25 | $27.29 | $53.07 | $36.48 | $36.48 |
| Vermont | $12.60 | $17.14 | $62.14 |  |  | $29.76 | $57.60 | $107.49 | $116.87 |
| Virginia | $14.96 | $19.95 | $79.81 | $79.74 | $122.99 | $22.55 | $43.95 | $104.54 | $123.50 |
| Washington | $11.54 | $15.70 | $56.92 | $104.82 | $143.44 | $22.88 | $44.22 | $81.80 | $90.60 |
| West Virginia | $12.60 | $17.14 | $62.14 | $114.43 | $156.59 | $25.91 | $50.75 | $103.37 | $128.80 |
| Wisconsin | $12.60 | $17.14 | $62.14 | $63.95 | $98.39 |  |  | $129.03 | $144.14 |
| Wyoming | $13.12 | $17.51 | $70.03 |  |  |  |  | $154.90 | $164.96 |
| Medicare | $12.60 | $17.14 | $62.14 | $114.43 | $156.59 | No coverage | No coverage | $180.75 | $201.68 |

Sources: Authors' analysis of Medicaid physician fees posted to state websites as of March 2021. Centers for Medicare & Medicaid Services (CMS). Physician Fee Schedule [Internet]. Baltimore (MD): CMS; 2021 [cited 2021 Aug 18]. Physician fee schedule data available from: https://www.cms.gov/Medicare/Medicare-Fee-for-Service-Payment/PhysicianFeeSched.

Notes: Fees are calculated as unweighted averages across provider types. $0 may denote that the service is not covered by the state or that the state uses provider-specific rates that they do not publish. Reported Medicare rates are national. Medicare has no national fees for alcohol and/or substance abuse structured SBI services (15 to 30 and >30 minutes). States covering a service includes $0 reports.

# **Appendix Table 2. Mean, 25th and 75th percentile of Medicaid and Medicare fees as of March 2021, by OTP methadone bundle type**

| **OTP methadone bundle type** | **Weighted mean fee ($) [a]** | **Unweighted 25th percentile fee ($)** | **Unweighted 75th percentile fee ($)** |
| --- | --- | --- | --- |
| Regular Methadone Bundle Fee Components, Medicaid (weekly) | 129.76 | 103.52 | 161.31 |
| Total Regular Methadone Bundle Fee and Additional Payments including care coordination, case management, opioid home health, and/or center of excellence services, Medicaid (weekly) | 154.44 | 111.51 | 207.49 |
| Medicare (weekly) | 210.34 | 202.40 | 213.92 |

Source: Authors' analysis of 2021 locality-specific payment rates for Opioid Treatment Programs and Medicaid physician fees posted to state websites as of March 2021. Centers for Medicare & Medicaid Services (CMS). Opioid Treatment Program [Internet]. Baltimore (MD): CMS; 2021 [cited 2021 Aug 18]. Opioid Treatment Program data available from: https://www.cms.gov/Medicare/Medicare-Fee-for-Service-Payment/Opioid-Treatment-Program/billing-payment.

Note: All fees in dollars. Fees are summary statistics for the Medicaid and Medicare reimbursement rates of the states included in the methadone index. Mean fees are raw averages across states; weighted mean fees are weighted by Medicaid/Medicare enrollment. The weekly bundle is based on the Medicare Opioid Treatment Program methadone weekly bundle fee component services of dispensing and/or administration, individual therapy (30 minutes), group therapy (30 minutes), toxicology testing, and care coordination and/or case management. See Table 1 for services included in each bundle type, by state.

[a] To estimate national average Medicaid and Medicare fees for each procedure, we weighted state fees by March 2021 Medicaid enrollment, which reflected the point-in-time number of nonelderly adult Medicaid enrollees not dually enrolled in Medicare in March 2021, estimated using factors computed from MSIS and CMS dual and child enrollment data.

# **Appendix Table 3. Mean, 25th and 75th percentile Medicaid fees as of March 2021, by procedure**

| **Code** | **Procedure** | **Index weight [a]** | **Weighted mean fee ($) [b]** | **Unweighted 25th percentile fee ($)** | **Unweighted 75th percentile fee ($)** |
| --- | --- | --- | --- | --- | --- |
| **Evaluation and Management** | | **25.00%** |  |  |  |
| 99212 | Ongoing evaluation and management, straightforward complexity | 8.33% | 30.47 | 29.01 | 41.13 |
| 99213 | Ongoing evaluation and management, low complexity | 8.33% | 46.02 | 40.84 | 66.86 |
| 99214 | Ongoing evaluation and management, medium complexity | 8.33% | 68.36 | 63.53 | 97.32 |
| **Psychotherapy** | | **25.00%** |  |  |  |
| 90832 | Psychotherapy, 30 minutes with patient and/or family member | 8.33% | 48.78 | 46.62 | 61.48 |
| 90834 | Psychotherapy, 45 minutes with patient and/or family member | 8.33% | 69.88 | 67.18 | 87.33 |
| 90853 | Group psychotherapy (other than multifamily) | 8.33% | 19.53 | 19.29 | 30.31 |
| **Toxicology testing** | | **25.00%** |  |  |  |
| 80305 | Testing for presence of drug, read by direct observation | 5.00% | 10.43 | 10.08 | 12.60 |
| 80306 | Testing for presence of drug, read by instrument assisted observation | 5.00% | 13.96 | 13.71 | 17.14 |
| 80307 | Testing for presence of drug, by chemistry analyzers | 5.00% | 50.19 | 49.71 | 62.14 |
| G0480 | Drug test, definitive 1-7 classes | 5.00% | 73.68 | 64.51 | 108.60 |
| G0481 | Drug test, definitive 8-14 classes | 5.00% | 116.60 | 104.54 | 156.59 |
| **Alcohol/substance use screening and psychiatric diagnostic evaluation** | | **25.00%** |  |  |  |
| 99408 | Alcohol and/or substance abuse structured SBI services; 15 to 30 minutes | 6.25% | 27.45 | 23.20 | 35.92 |
| 99409 | Alcohol and/or substance abuse structured SBI services; > 30 minutes | 6.25% | 51.54 | 45.96 | 67.84 |
| 90791 | Psychiatric diagnostic evaluation | 6.25% | 110.82 | 103.37 | 131.10 |
| 90792 | Psychiatric diagnostic evaluation with medical services | 6.25% | 120.11 | 105.37 | 148.18 |

Source: Authors' analysis of Medicaid physician fees posted to state websites as of March 2021.

Note: All fees in dollars. Fees are summary statistics for the estimated Medicaid reimbursement rates of the states included in the Other Services index. Mean fees are raw averages across states; weighted mean fees are weighted by Medicaid enrollment. Index weights are the weights applied to the Medicaid national average ratios for each procedure in the indexes; each group of procedures has equal weight.

[a] For the index of services related to OUD treatment, we created a state-level Medicaid fee index and respective sub-indexes for the four subgroups (evaluation and management, psychotherapy, toxicology testing, and substance use screening and diagnostic evaluation) using equal weights for the codes within each of the four subgroups, as shown.

[b] To estimate national average Medicaid and Medicare fees for each procedure, we weighted state fees by March 2021 Medicaid enrollment, which reflected the point-in-time number of nonelderly adult Medicaid enrollees not dually enrolled in Medicare in March 2021, estimated using factors computed from MSIS and CMS dual and child enrollment data.

# **Appendix Table 4. Mean, 25th and 75th percentile Medicare fees as of March 2021, by procedure**

| **Code** | **Procedure** | **Index weight [a]** | **Weighted mean fee ($) [b]** | **Unweighted 25th percentile fee ($)** | **Unweighted 75th percentile fee ($)** |
| --- | --- | --- | --- | --- | --- |
| **Evaluation and Management** | | **25.00%** |  |  |  |
| 99212 | Ongoing evaluation and management, straightforward complexity | 8.33% | 56.27 | 53.37 | 57.61 |
| 99213 | Ongoing evaluation and management, low complexity | 8.33% | 91.59 | 87.31 | 93.75 |
| 99214 | Ongoing evaluation and management, medium complexity | 8.33% | 130.04 | 124.17 | 132.91 |
| **Psychotherapy** | | **25.00%** |  |  |  |
| 90832 | Psychotherapy, 30 minutes with patient and/or family member | 8.33% | 77.81 | 75.78 | 78.30 |
| 90834 | Psychotherapy, 45 minutes with patient and/or family member | 8.33% | 103.19 | 100.32 | 103.86 |
| 90853 | Group psychotherapy (other than multifamily) | 8.33% | 27.55 | 26.79 | 27.75 |
| **Toxicology testing** | | **25.00%** |  |  |  |
| 80305 | Testing for presence of drug, read by direct observation | 5.00% | 12.60 | 12.60 | 12.60 |
| 80306 | Testing for presence of drug, read by instrument assisted observation | 5.00% | 17.14 | 17.14 | 17.14 |
| 80307 | Testing for presence of drug, by chemistry analyzers | 5.00% | 62.14 | 62.14 | 62.14 |
| G0480 | Drug test, definitive 1-7 classes | 5.00% | 114.43 | 114.43 | 114.43 |
| G0481 | Drug test, definitive 8-14 classes | 5.00% | 156.59 | 156.59 | 156.59 |
| **Alcohol/substance use screening and psychiatric diagnostic evaluation** | | **25.00%** |  |  |  |
| 99408 | Alcohol and/or substance abuse structured SBI services; 15 to 30 minutes | 6.25% | 36.06 | 34.67 | 36.54 |
| 99409 | Alcohol and/or substance abuse structured SBI services; > 30 minutes | 6.25% | 69.35 | 66.70 | 70.30 |
| 90791 | Psychiatric diagnostic evaluation | 6.25% | 180.56 | 175.48 | 181.93 |
| 90792 | Psychiatric diagnostic evaluation with medical services | 6.25% | 201.36 | 195.43 | 203.15 |

Sources: Authors' analysis of 2021 Q1 Medicare physician fee schedule and clinical laboratory fee schedule. Centers for Medicare & Medicaid Services (CMS). Physician Fee Schedule [Internet]. Baltimore (MD): CMS; 2021 [cited 2021 Aug 18]. Physician fee schedule data available from: https://www.cms.gov/Medicare/Medicare-Fee-for-Service-Payment/PhysicianFeeSched. CMS. Clinical Laboratory Fee Schedule [Internet]. Baltimore (MD): CMS; 2021 [cited 2021 Aug 18]. Clinical laboratory fee schedule data available from: https://www.cms.gov/Medicare/Medicare-Fee-for-Service-Payment/ClinicalLabFeeSched.

Note: All fees in dollars. Fees are summary statistics for the Medicare reimbursement rates of the states included in the Other Services index. Medicare reimbursement rates are calculated using relative value units, geographic adjusters, and conversion factors obtained from CMS. Toxicology testing fees are paid based on a national laboratory services fee schedule and do not differ across states. Mean fees are raw averages across states; weighted mean fees are weighted by Medicare enrollment. Index weights are the weights applied to the Medicaid-Medicare ratios for each procedure in the indexes; each group of procedures has equal weight.

[a] For the index of services related to OUD treatment, we created a state-level Medicaid fee index and respective sub-indexes for the four subgroups (evaluation and management, psychotherapy, toxicology testing, and substance use screening and diagnostic evaluation) using equal weights for the codes within each of the four subgroups, as shown.

[b] To estimate national average Medicaid and Medicare fees for each procedure, we weighted state fees by March 2021 Medicaid enrollment, which reflected the point-in-time number of nonelderly adult Medicaid enrollees not dually enrolled in Medicare in March 2021, estimated using factors computed from MSIS and CMS dual and child enrollment data.

# **Appendix Table 5. Medicaid-to-Medicare fee index for selected common services, percent of Medicaid enrollees treated for opioid use disorder (OUD), non-dually enrolled Medicaid enrollees aged 12-64, and percent of Black, non-Hispanic Medicaid enrollees aged 12-64, by state, with Spearman's rank correlation coefficients**

|  | **All services Medicaid-to-Medicare fee index, 2019** | **Medicaid enrollees treated for OUD, 2019** | **Medicaid enrollment, 2021** | **Non-Hispanic, Black Medicaid enrollment, 2019** |
| --- | --- | --- | --- | --- |
| Spearman's Rho | 0.35 | -0.16 | -0.12 | -0.05 |
| Alabama | 0.84 | 1.9% | 230,009 | 41.3% |
| Alaska | 1.10 | 3.8% | 124,662 | 2.9% |
| Arizona | 0.82 | 3.6% | 1,698,567 | 6.5% |
| Arkansas | 0.79 | 0.7% | 446,141 | 23.4% |
| California | 0.73 | 1.6% | 5,963,559 | 7.6% |
| Colorado | 0.81 | 2.6% | 811,464 | 6.7% |
| Connecticut | 0.75 | 5.5% | 531,666 | 18.1% |
| Delaware | 1.18 | 6.9% | 130,637 | 31.1% |
| District of Columbia | 0.80 | 2.0% | 143,321 | 75.4% |
| Florida | 0.58 | 1.4% | 1,276,046 | 23.8% |
| Georgia | 0.83 | 1.1% | 517,746 | 47.8% |
| Hawaii | 0.62 | 1.3% | 204,646 | 0.7% |
| Idaho | 0.93 | 2.5% | 160,825 | 1.9% |
| Illinois | 0.59 | 1.9% | 1,463,850 | 27.4% |
| Indiana | 0.77 | 3.7% | 779,284 | 17.5% |
| Iowa | 0.81 | 1.7% | 319,867 | 9.5% |
| Kansas | 0.71 | 1.2% | 101,708 | 11.6% |
| Kentucky | 0.76 | 5.5% | 835,289 | 10.9% |
| Louisiana | 0.69 | 2.3% | 810,469 | 47.8% |
| Maine | 0.66 | 6.7% | 149,938 | 3.3% |
| Maryland | 0.90 | 7.1% | 691,864 | 43.3% |
| Massachusetts | 0.78 | 6.2% | 747,307 | 11.6% |
| Michigan | 0.69 | 2.6% | 1,454,495 | 24.9% |
| Minnesota | 0.74 | 3.1% | 546,363 | 14.2% |
| Mississippi | 0.92 | 1.1% | 171,895 | 59.9% |
| Missouri | 0.62 | 2.3% | 315,722 | 22.5% |
| Montana | 1.11 | 3.4% | 133,150 | 0.8% |
| Nebraska | 1.05 | 0.9% | 100,013 | 9.2% |
| Nevada | 0.96 | 2.7% | 394,027 | 17.8% |
| New Hampshire | 0.57 | 6.8% | 96,028 | 1.3% |
| New Jersey | 0.50 | 2.5% | 816,649 | 20.0% |
| New Mexico | 0.93 | 5.5% | 413,155 | 2.0% |
| New York | 0.57 | 2.7% | 3,354,637 | 20.1% |
| North Carolina | 0.78 | 2.6% | 542,565 | 36.0% |
| North Dakota | 1.00 | 1.2% | 51,258 | 4.0% |
| Ohio | 0.62 | 5.5% | 1,464,011 | 24.2% |
| Oklahoma | 0.94 | 2.3% | 207,786 | 13.6% |
| Oregon | 0.83 | 3.9% | 578,633 | 3.1% |
| Pennsylvania | 0.68 | 5.4% | 1,508,137 | 20.2% |
| Rhode Island | 0.37 | 5.6% | 163,617 | 10.5% |
| South Carolina | 0.98 | 1.5% | 351,574 | 46.7% |
| South Dakota | 0.85 | 1.0% | 28,443 | 2.7% |
| Texas | 0.65 | 0.7% | 874,774 | 17.8% |

**Appendix Table 5 (continued). Medicaid-to-Medicare fee index for selected common services, percent of Medicaid beneficiaries treated for opioid use disorder (OUD), non-dually enrolled Medicaid enrollees aged 12-64 and percent of Black, non-Hispanic Medicaid enrollees aged 12-64, by state, with Spearman's rank correlation coefficients**

|  | **All services Medicaid-to-Medicare fee index, 2019** | **Percent of Medicaid enrolees treated for OUD, 2019** | **Medicaid enrollment, 2021** | **Non-Hispanic, Black Medicaid enrollment, 2019** |
| --- | --- | --- | --- | --- |
| Utah | 0.81 | 4.4% | 148,202 | 3.2% |
| Vermont | 0.86 | 7.9% | 95,038 | 1.3% |
| Virginia | 0.78 | 3.1% | 689,255 | 32.5% |
| Washington | 0.69 | 4.6% | 954,155 | 6.5% |
| West Virginia | 0.71 | 6.1% | 295,723 | 4.9% |
| Wisconsin | 0.62 | 3.4% | 539,585 | 18.1% |
| Wyoming | 0.96 | 1.4% | 18,325 | 3.0% |

Source: Authors' analysis of 2019 American Community Survey data and monthly Medicaid enrollment data, monthly Medicare-Medicaid dual enrollment data, and MSIS claims data posted to state websites as of March 2021. Prior Urban analysis of Medicaid physician fees posted to state websites as of September 2019. 2014 Medicaid Statistical Information System (MSIS). Centers for Medicare & Medicaid Services (CMS). Monthly Enrollment Data [Internet]. Baltimore (MD): CMS; 2021 [cited 2021 Aug 26]. Enrollment data available from: <https://www.medicaid.gov/medicaid/national-medicaid-chip-program-information/medicaid-chip-enrollment-data/monthly-medicaid-chip-application-eligibility-determination-and-enrollment-reports-data/index.html>. Dual enrollment data available from: <https://www.cms.gov/Medicare-Medicaid-Coordination/Medicare-and-Medicaid-Coordination/Medicare-Medicaid-Coordination-Office/Analytics>. Medicaid OUD treatment rates available from: https://portal.cms.gov/MSTR10Prd/servlet/mstrWeb?src=mstrWeb.2048001&ru=1&documentID=E8E899A911EB08AB795A0080EFE5D251&evt=2048001&share=1&hiddensections=header%2Cpath%2CdockTop%2CdockLeft%2Cfooter&Server=V343069P&Port=0&Project=SUD+Data+Book_Prd&.

Note: The all services index, from previously published computations, was calculated from Medicaid fees for twenty-seven common procedures, including primary care, obstetrical care, and other services, on state websites as of September 2019.(5) Medicaid enrollment reflects the point-in-time number of nonelderly adult Medicaid enrollees not dually enrolled in Medicare in March 2021, estimated using factors computed from MSIS and CMS dual and child enrollment data. Spearman's rho rank correlation coefficients measure the association between the Medicaid-to-Medicare index for the regular methadone bundle (Table 2) and the Medicaid-to-Medicare index for selected common services, OUD treatment rates, Medicaid enrollment, and non-Hispanic, Black Medicaid enrollment, respectively.
